# The *Australian Shark-Incident Database* for quantifying temporal and spatial patterns of shark-human conflict

**DOI:** 10.1038/s41597-022-01453-9

**Published:** 2022-07-06

**Authors:** Madeline Riley, Phoebe Meagher, Charlie Huveneers, Jacob Leto, Victor M. Peddemors, David Slip, John West, Corey J. A. Bradshaw

**Affiliations:** 1 Southern Shark Ecology Group, College of Science and Engineering, Flinders University, GPO Box 2100, Adelaide, South Australia 5001 Australia; 2 Global Ecology Partuyarta Ngadluku Wardli Kuu, College of Science and Engineering, Flinders University, GPO Box 2100, Adelaide, South Australia 5001 Australia; 3 Taronga Institute of Science and Learning, Taronga Conservation Society Australia, Mosman, New South Wales 2088 Australia; 4 NSW Department of Primary Industries, Fisheries, Sydney Institute of Marine Science, Mosman, Sydney, NSW 2088 Australia

**Keywords:** Animal behaviour, Behavioural ecology, Environmental social sciences

## Abstract

We describe the *Australian Shark-Incident Database*, formerly known as the *Australian Shark-Attack File*, which contains comprehensive reports of 1,196 shark bites that have occurred in Australia over 231 years (1791–2022). Data were collated by the Taronga Conservation Society Australia using purpose-designed questionnaires provided to shark-bite victims or witnesses, media reports, and information provided by the department responsible for fisheries in each Australian state (including the Northern Territory). The dataset includes provoked and unprovoked bites from fresh, brackish, and marine waters in Australia. Data span 22 suspected shark species. This dataset will be publicly available, and can be used by analysts to decipher environmental, biological, and social patterns of shark bites in Australia. The information will aid scientists, conservationists, authorities, and members of the public to make informed decisions when implementing or selecting mitigation measures.

## Abstract (summary)

**Table:** 

Measurement(s)	Shark bite incidents
Technology Type(s)	survey
Factor Type(s)	none
Sample Characteristic - Organism	Elasmobranchii
Sample Characteristic - Environment	ocean
Sample Characteristic - Location	Australia

## Background & Summary

Globally, shark bites on humans have increased steadily over the past four decades^1,2^. However, shark bites are decreasing in some regions and remain stable in others, reflecting the high variability of the risk of being bitten by a shark^2,3^. Between 1982 and 2011, 84% of reported shark bites were in only six countries (United States, Australia, South Africa, Brazil, France (Réunion Island), and the Bahamas). Of these countries, Australia had the second-highest number of total bites^1^, which has risen from an average of nine bites year^−1^ from 1990–2000 to 22 bites year^−1^ from 2010–2020.

Potential causes behind the rise in shark bites in Australia are still debated. The foremost hypothesis for the recent rise in shark bites is human population growth, particularly along the coast^4,5^ and increased time spent doing water-based activities such as surfing and diving^4–8^. Environmental and habitat variation, such as changing water temperature^6^, decreased water clarity^9^, and climate change^10^ are also likely to contribute to changes in the number of shark bites and the increased occurrences in some regions^11,12^. The low and sporadic number of bite incidents and complexity of factors influencing shark-bite risk underline the challenges of understanding their drivers and what might cause sharks to bite humans.

Records of shark bites in Australia have been kept since European settlement, although the reliability of earlier records is not easily verified. The *Australian Shark-Incident Database* (formerly known as the *Australian Shark-Attack File)* is considered the principal source of shark-bite data in Australia^5,13^. The *Australian Shark-Attack File* was founded by John West in the 1980s and based on the initial research by David Baldridge, whose 1974 analysis of the database resulted in the first book on shark-human interactions. The change in the database name presented here reflects negative concerns associated with the term ‘attack’ and more accurately describes the behaviours and outcomes of shark-human interactions in a less inflammatory way^14^.

The database has been maintained by Taronga Conservation Society Australia (hereafter referred to as Taronga), since 1984 and includes 1,196 individual investigations of shark-bite cases in Australia between 1791–2022. Prior to being entered into the database, all reported shark-bite cases are subject to thorough assessment to collate all possible information about incidents. The database is updated as new information becomes available. Gathering this information enables a comprehensive evaluation of the context and potential causes of shark bites and could assist in reducing these incidents and promoting sustainable shark-human coexistence.

Detailed information about shark-bite incidents can reveal patterns and trends that enhance our understanding of the potential drivers of the probability of being bitten by a shark^5,15,16^. In turn, this information can be used to predict or avoid future shark bites^12,17^. For example, Bradshaw *et al*.^18^ used the database combined with known deterrent effectiveness to predict the potential future reduction in shark bites on people in Australia. The data can also enable policy makers and consumers to make informed decisions when selecting and implementing the most appropriate shark-bite mitigation measures for the intended outcome^19^. For example, by assessing the most common activity at the time of a shark bite (e.g., surfing, swimming, diving), developing and implementing mitigation strategies can focus on higher-risk activities. Further, analysts can use these data to assess long-term shifts in species distributions resulting from phenomena such as climate change^17^.

Here, we describe and present the *Australian Shark-Incident Database*, including a protocol to standardise future database applications and account for quality assurance and control. This database is now available publicly and can be used to explore patterns and trends in shark incidents across Australia, ultimately improving our understanding of the causes and consequences of these incidents and to avoid or predict events in the future^20^.

## Methods

### Data acquisition

The *Australian Shark-Incident Database* master file is a dedicated database securely stored on Taronga’s ELO Java Client content-management database and only accessible to edit by Taronga’s dedicated team. The database is the most comprehensive dataset of shark-bite incidents in Australia. The database has been maintained by Taronga since 1984. Prior to 1984, records of shark-bite incidents dating back to 1791 were collated by the founder of the database, John West, using historical media reports, books, government reports, victim, and witness accounts. The original dataset is a comprehensive repository with 100 descriptor fields including information such as geographical location of the incident, weather conditions, victim recovery status, shark species implicated, and time of incident.

**Fig. 1:**
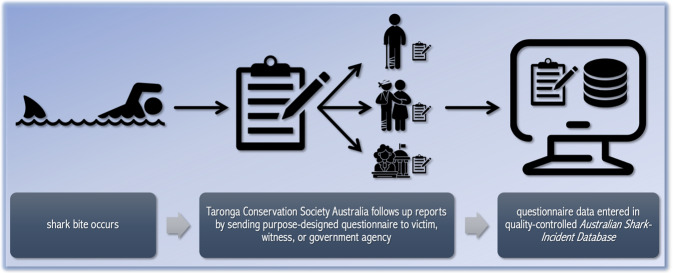
Flow chart of data acquisition used to collate the *Australian Shark-Incident Database*.

The database is now updated as close to the time of the incident as is possible. Initial data are drawn from media reports manually and followed up by Taronga with a purpose-designed questionnaire (Supplementary File 1), sent to either the victim, a witness, or the state (or territory; hereafter referred to as State) department responsible for fisheries within which the incident occurred. Participants’ consent to use the data was obtained by completing and signing the questionnaire which includes a statement notifying that information provided may be used for research purposes. Upon receiving the completed questionnaire, data are reviewed by trained staff and information is validated before populating the master database (Fig. 1). Since 2008, the New South Wales Department of Primary Industries have developed a more detailed questionnaire that includes all ASID data fields and is sent to Taronga to populate the national database. All supporting questionnaires, files, documents, photos, and links to media are also stored securely and restricted in access to ensure that users comply with ethical requirements.

All reports of a shark bite in Australia are assessed against the following criteria for inclusion. Shark-human interactions should be included in the database if they meet all of the following three criteria: (*i*) the person is alive at the time of the incident, (*ii*) the person is in the water or using a small watercraft (e.g., kayak, surfboard, bodyboard, non-motorised vessel), and (*iii*) there is a determined attempt by a shark to bite a person, equipment worn or being used, or the small watercraft. For the purpose of this database, the interaction should be recorded regardless of whether the bite is successful (i.e., occurs).

Shark experts in the relevant State’s department responsible for fisheries are contacted if species identification or confirmation is required. Where appropriate, the State department will liaise with emergency services as well as forensically investigate any bite marks and teeth that might have been recovered using a broad range of methods^21–25^. Once species validation has occurred, the incident is reported on the Taronga website (https://taronga.org.au/conservation-and-science/australian-shark-incident-database). In some cases, the species responsible for the incident is unknown and is not included in the record. Only some summary data are presented on the website. Taronga chooses to present only unprovoked data by jurisdiction and injury type, and an overall total including provoked cases.

An ‘unprovoked’ encounter between a human and a shark is defined as an incident where a shark is in its natural habitat and has made a determined attempt to bite a human where that person is not engaged in provocative activities. A ‘provoked’ incident relates to circumstances where the person attracts or initiates physical contact with a shark (accidentally or on purpose) or was fishing for, stabbing, feeding, netting, or handling a shark, or where the shark was attracted to the victim by activities such as fishing, spearfishing (where a fish has already been speared), and cleaning of captured fish. Until 2021, all incidents involving a spearfisher or a commercial diver were categorised as ‘provoked’. Since review of provocation criteria, incidents involving spearfishers will now be classed as ‘provoked’ only if a fish has already been speared. Incidents involving rays are considered out of scope and are not included in the database.

### Computational processing

While the *Australian Shark-Incident Database* is the most comprehensive record of Australian shark-bite cases, we enhanced aspects of the database to allow for broader applications. Prior to this publication, the complete database was not publicly available unless data were specifically requested. There were also inconsistencies within the database that made it difficult to compare between cases, such as inconsistent naming conventions and descriptive entries rather than categorical classifications. To account for these issues, we developed a protocol (Supplementary File 2) for updating and maintaining the database to account for quality assurance and control (outlined in *Data Records*). We updated the data in accordance with the protocol using Microsoft Excel.

## Data Records

The *Australian Shark-Incident Database* (10.5281/zenodo.5612259^26^)contains 1,196 unique shark-bite records between 01/01/1791 until 08/03/2022. All columns in the database containing personal or sensitive information are not visible to the public to retain confidentiality for the victims and their families. Periodic updates will be uploaded to the online database to allow for ongoing public access.

Each database row contains information relating to a unique shark-bite incident. The database includes 71 columns reporting information relative to each shark-bite incident (Supplementary File 2). The columns are individual fields that are purpose-designed to build an overview of the incident by gaining information such as geographical location where the bite occurred (e.g., state, latitude, longitude, and site description), shark species implicated (e.g., scientific name, identification method, identification source, and body length of shark implicated), context of bite (e.g., victim activity, attractant, or victim clothing), injuries sustained (e.g., injury location and severity), and environmental conditions (e.g., water visibility, sea condition, and tidal cycle). Each field is accompanied by a description of the information to be included in that section. Fields are also assigned a format so that the data remain consistent for analysis and quality control. The data fields can be numeric, descriptive, or categorical. Numeric fields are further defined as either integer, year, metres, or time, including specification for units (e.g., degrees Celsius, decimal degrees). We provide a full description of each column in Supplementary File 2.

## Technical Validation

There are two phases involved in supporting the technical quality of the dataset: (*i*) the process used by Taronga when collecting data for each shark-bite incident, and (*ii*) the consistency modifications that we made during manuscript development.

### Phase one

For each shark-bite incident, Taronga attempts to contact the most-relevant person involved in the event as possible — e.g., victim, victim’s family, witnesses. Those contacted are asked to complete a questionnaire with information relating to the shark bite. If applicable, questionnaires can also be completed by a fisheries officer in the relevant State and sent to Taronga. Taronga works closely with experts in each State’s fisheries department to validate information sourced in media reports. Each shark-bite case is unique, so the validation process varies depending on details specific to each incident. Forensic shark scientists within each State department are contacted after each shark bite to confirm details related to the incident. For example, validating species responsible for the bite often requires forensic analysis through expert examination of bite marks or artefacts^21–25^. When available, video footage is analysed for validation of information, such as confirmation of shark species and length.

The database is cross-checked annually for the previous year with the *International Shark-Attack File* in Florida, USA, as well as with fisheries officers to ensure consistency. The database is cross-checked with the acknowledgement that there are discrepancies between versions due to differences in inclusion criteria. For example, the *International Shark-Attack File* includes bites where the victim was bitten aboard a boat, whereas the database we present here does not include bites aboard a boat.

We acknowledge the limitations associated with this database, such as differences in reporting over time. For example, incidents might be reported more in recent decades due to technological advances making reporting more accessible or media publicising these events more widely^18^. There might also be reporting biases, for example, victims could be more likely to report a bite by a large, potentially dangerous shark (e.g., white, tiger, or bull shark) rather than a smaller, less-dangerous shark species (e.g., wobbegong shark). We also completed a quality assessment of the original database fields and redesigned the data acquisition and entry process (see *Phase two*) to allow exploration of shark-bite trends and patterns in Australia.

### Phase two

We identified errors and inconsistencies in database fields. To avoid additional errors and inconsistencies, and to obtain a quality-controlled database, we redesigned the process for gaining and entering information into the database. This included creating a data descriptor (Supplementary File 2) used as a protocol to inform which questions to ask in the questionnaire. The data descriptor also directs the format of database entries by specifying information required in each field and by indicating the format of each entry (i.e., numeric, descriptive, or categorical). We manually inspected all previously entered data and adapted them to match the data descriptor. We checked each entry using the filter function in Microsoft Excel to identify any spelling and grammatical errors in the fields and ensure that all categories were grammatically identical. We standardised all metrics during this process (e.g., the data descriptor now stipulates that all length measurements should be recorded in metres).

**Fig. 2:**
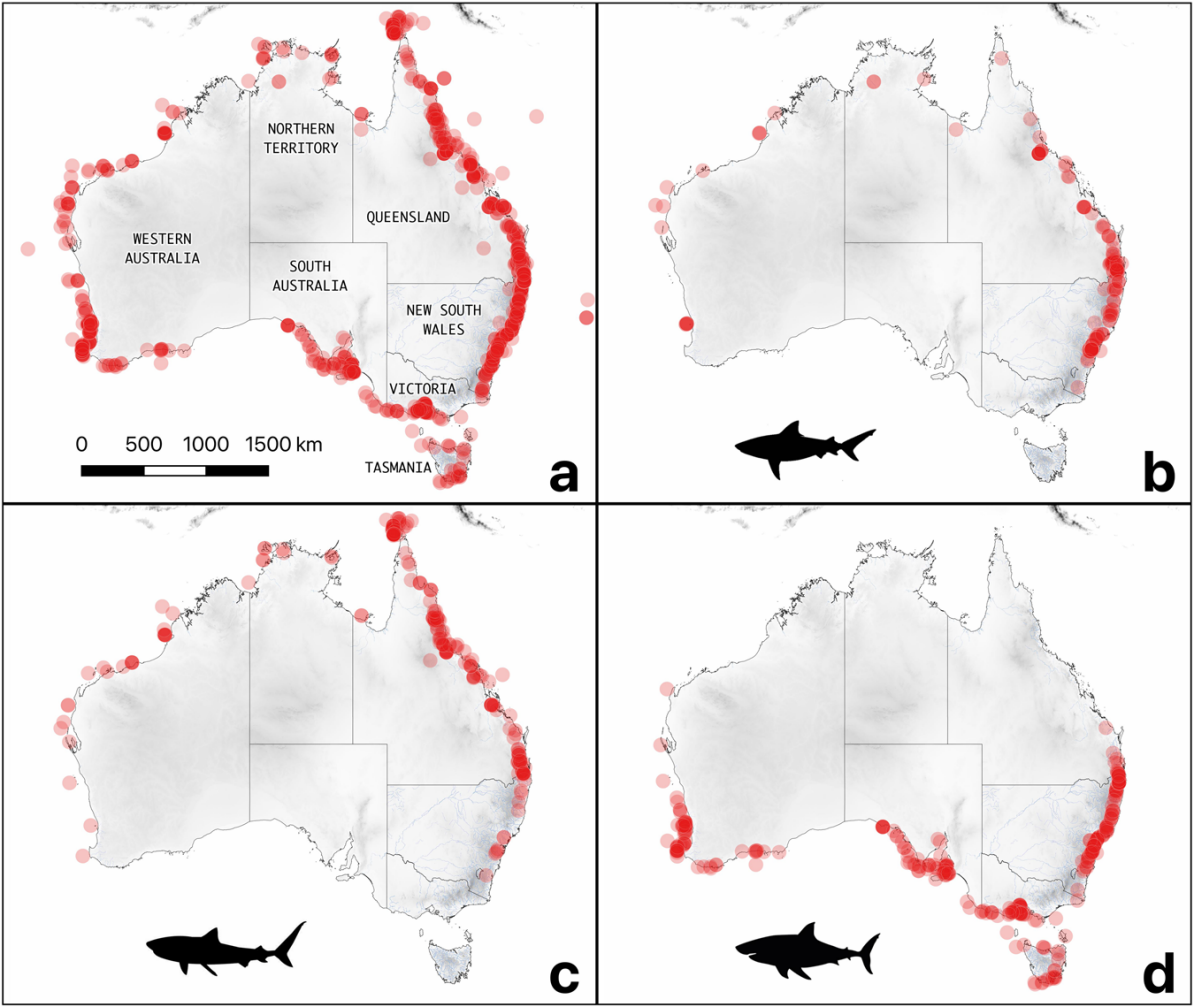
Geographical locations of 1,196 shark bites in Australia. Each shark-bite incident is indicated by a red dot. (**a**) all shark-bite incidents; (**b**) bites most likely inflicted by bull sharks, (**c**) tiger sharks, and (**d**) white sharks. Two bite incidents that occurred at the Australian external territory, Cocos (Keeling) Islands, are not included on these maps. Background layers show elevation and major, perennial watercourses.

We validated and standardised the geographical locations of shark bites by converting all coordinates into decimal degrees using Microsoft Excel. We subsequently plotted all coordinates using the ggmap library^27^ in R (Version 4.0.2) (R Core Team 2020) (Fig. 2). We corrected any unusual coordinates (e.g., outside of Australian waters) and crosschecked them with site descriptions and states to ensure validity.

**Fig. 3:**
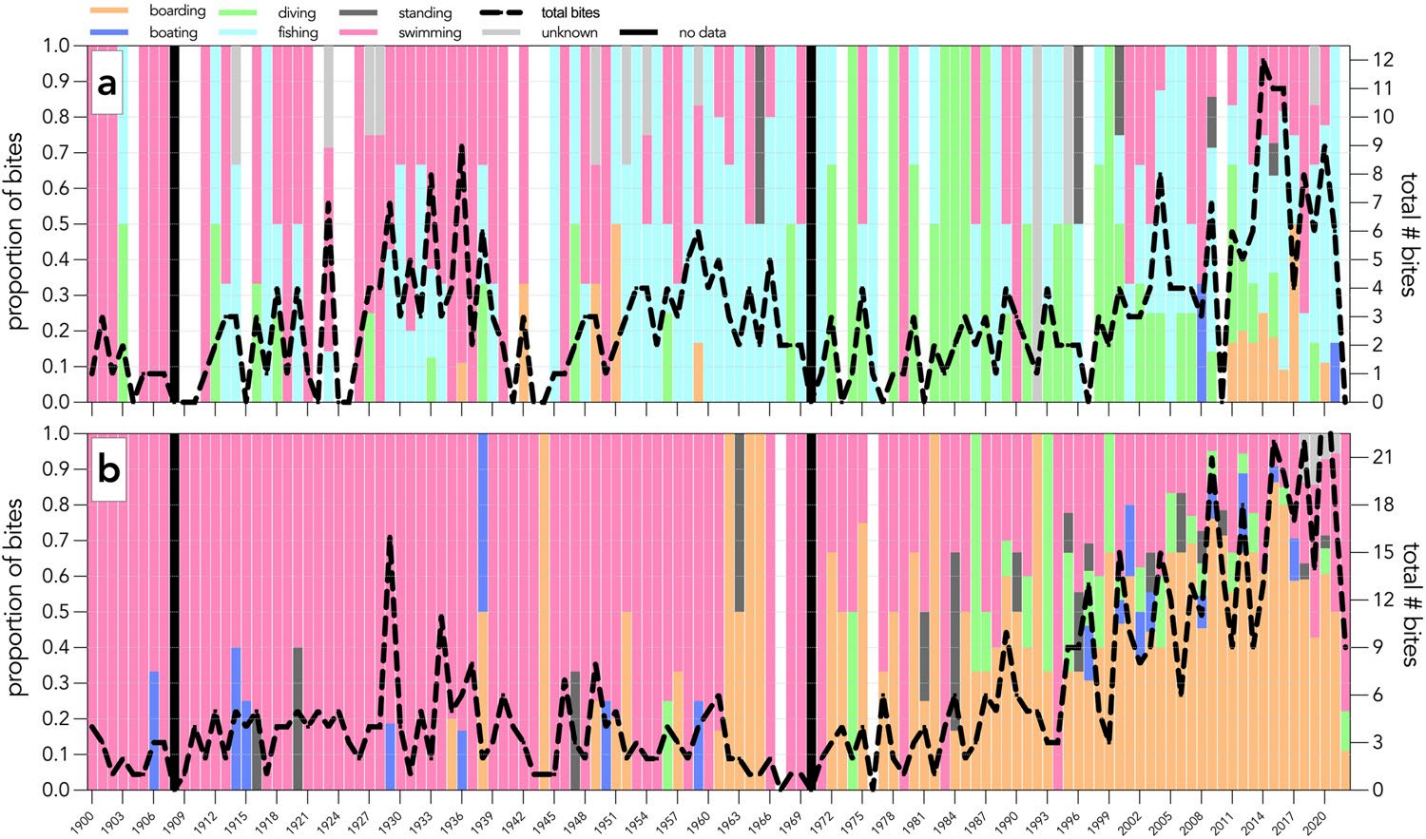
Number of shark bites (black, dashed line) and proportion of activity done by the victim at the time of shark-bite incidents in Australia from 1900 to 2022. Panels represent shark bites in Australia that are; (**a**) provoked or (**b**) unprovoked. Boarding includes surfboarding, bodyboarding, kiteboarding, sailboarding, wakeboarding, and stand-up paddle boarding. Swimming includes snorkelling, spearfishing, freediving, body surfing, clinging to an object, falling into water, floating, or wading. Diving includes scuba-diving, hookah diving, or hard-hat diving. Fishing includes cleaning fish. No data for years 1908 and 1970.

**Fig. 4:**
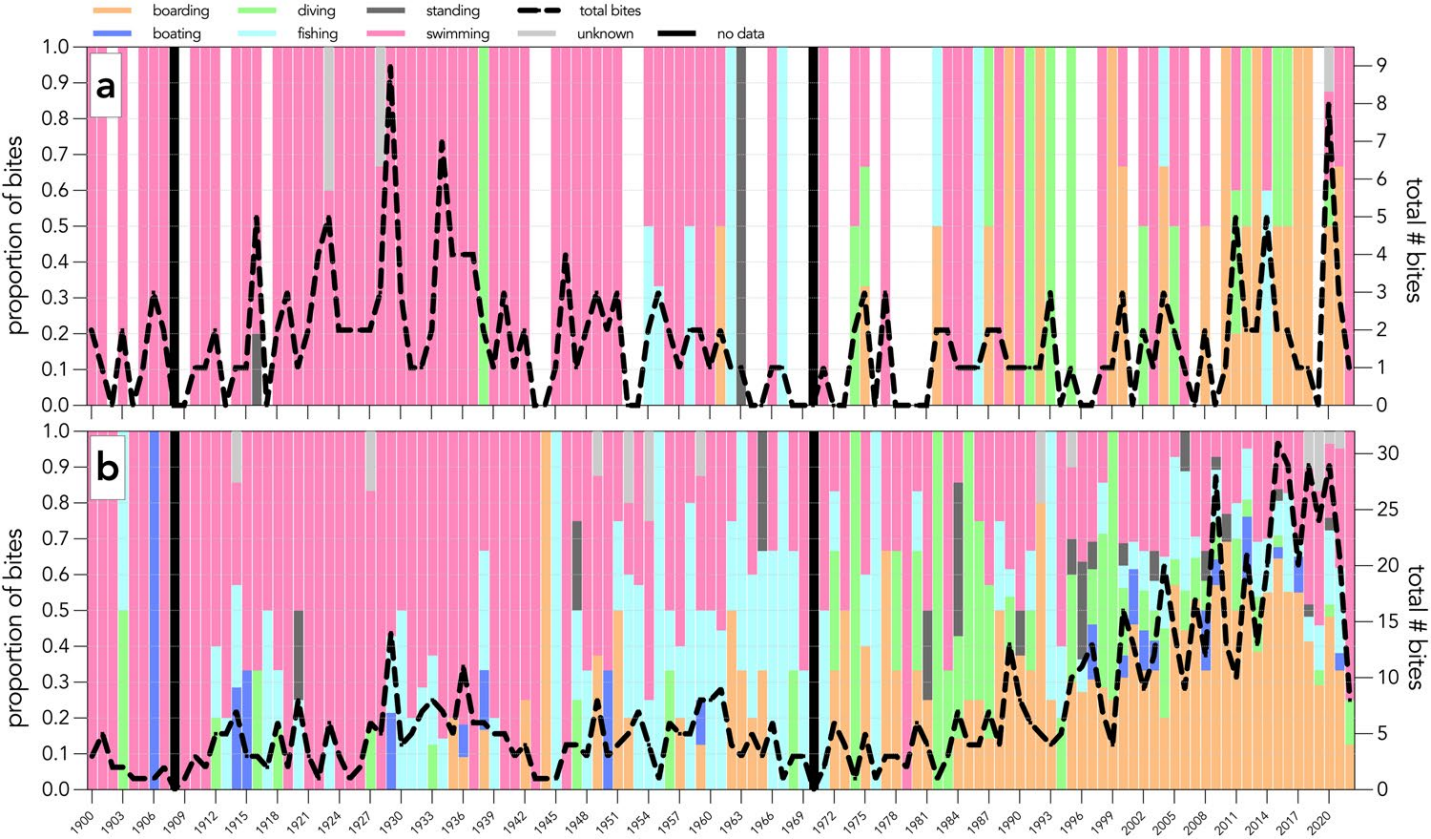
Number of shark bites (black, dashed line) and proportion of activity done by the victim at the time of shark-bite incidents in Australia from 1900 to 2022. Panels represent shark bites in Australia that are; (**a**) fatal *or* (**b**) non-fatal. Boarding includes surfboarding, bodyboarding, kiteboarding, sailboarding, wakeboarding, and stand-up paddle boarding. Swimming includes snorkelling, spearfishing, freediving, body surfing, clinging to an object, falling into water, floating, or wading. Diving includes scuba-diving, hookah diving, or hard-hat diving. Fishing includes cleaning fish. No data for years 1908 and 1970.

During the development of the data-descriptor protocol, we converted some previous descriptive columns into categorical columns. These columns included (but are not limited to) *victim activity*, *attractant*, *injury location*, *injury severity*, and *weather condition*. Converting these columns into categories facilitates analysis to investigate shark-bite patterns. For example, we converted *victim activity* into a categorical field to restrict answers to the following: *snorkelling*, *motorised boating*, *unmotorised boating*, *boarding*, *swimming*, *standing*, *diving*, *fishing*, or *other*, rather than allowing answers in any format. We used this information to create a time series to show the activity of shark-bite victims in Australia over time (Figs. 3 and 4). Shark bites have increased for boarders (including surfboarding, bodyboarding, kiteboarding, sailboarding, wakeboarding, and stand-up paddle boarding) over time, particularly since 1960 (Figs. 3b, 4). This is likely due to the increase in popularity of board sports, particularly surfing, since the 1960s^28^. This trend is likely not reflected in Fig. 3a because shark bites are unlikely to be classed as ‘provoked’ during board riding.

**Fig. 5:**
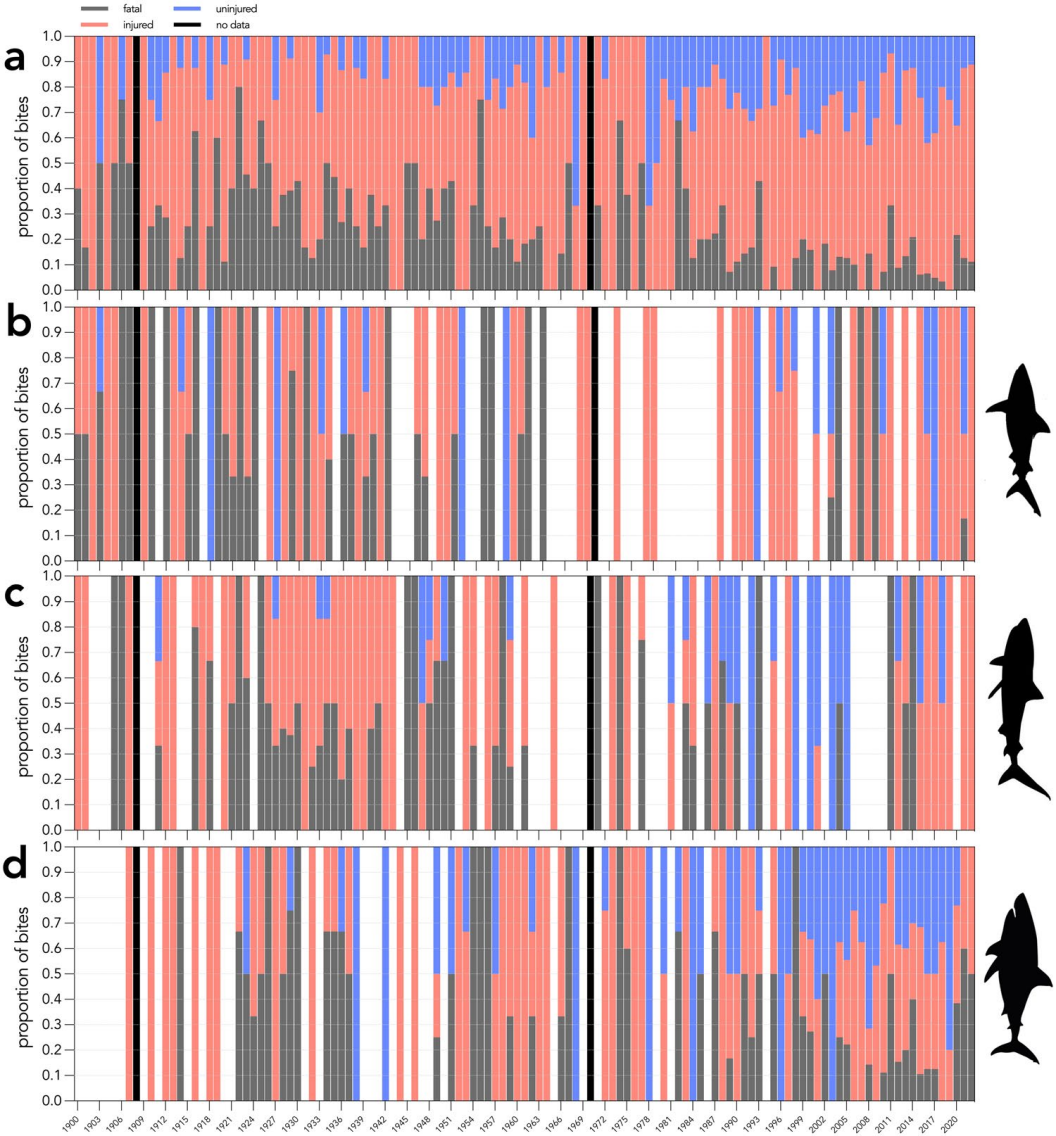
Proportion of victim-recovery status (fatal = grey; red = injured; blue = uninjured) resulting from all unprovoked shark bites in Australia between 1900–2022. Blank years represent years without any reported occurrences. (**a**) all shark-bite incidents, (**b**) bites most likely inflicted by bull sharks, (**c**) tiger sharks, or (**d**) white sharks. No data for years 1908 and 1970.

The column representing a shark-bite victim’s *recovery status* also requires a categorical response, restricting answers to: *fatal, injured, or uninjured* (Fig. 5). Since 1900, the proportion of shark-bite-related fatalities has decreased (Fig. 5). This trend is also true for the three species most attributed to shark-bite-related fatalities, white (*Carcharodon carcharias*), tiger (*Galeocerdo cuvier*), and bull (*Carcharhinus leucas*) sharks. The decrease in shark-bite-related deaths is likely due to advancements in medical responses to shark-bite victims over time^14^ and better understanding among surfers about using tourniquets to stem bleeding following increased certification as first responders in workplace occupational health and safety requirements. Bites resulting in an *uninjured* victim includes interactions where the shark might have bitten the victim’s equipment (i.e., surfboard, bodyboard, kayak) rather than biting the person.

**Fig. 6:**
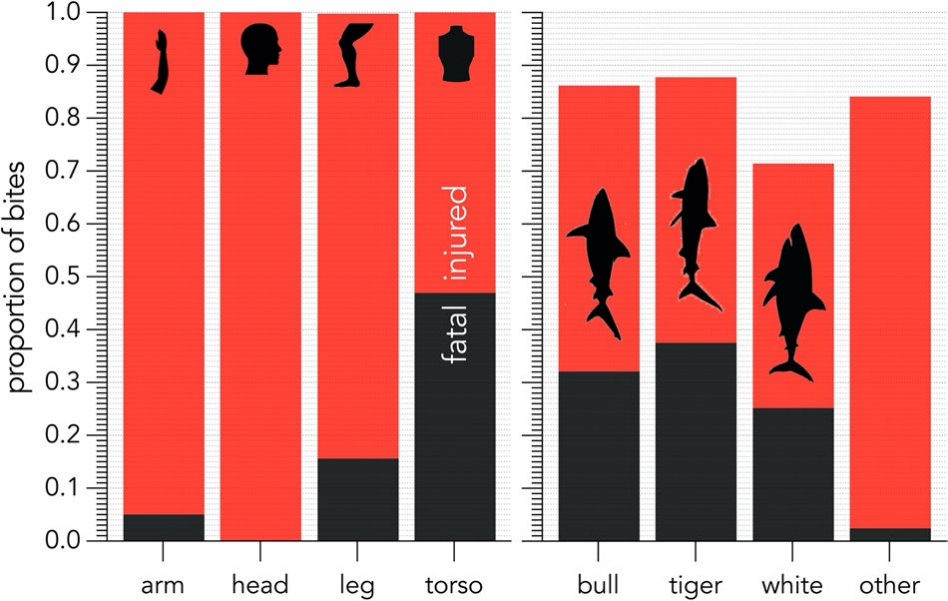
Proportion of Australian shark bites resulting in either fatality or injury categorised by injury location on the victim’s body (left panel; 250 bites resulting in fatal injury, 723 bites resulting in non-fatal injury) and by species (right panel; bites by 201 tiger, 170 bull, 258 white, and 303 bites other sharks).

Previously, entries in the *injury location* column were descriptive. We converted the column to a categorical field restricting answers to the following: *arm*, *hand*, *lower arm*, *upper arm*, *shoulder*, *neck*, *head*, *torso*, *leg*, *foot*, *calf*, *thigh*, *pelvic region*, or *other*. During the analyses process, we further categorised these injury locations into four body areas (*head, arm, torso*, and *leg*) to assess how injury location affects recovery status (fatal or injured) (Fig. 6). Fatality most often occurred following shark bites to the torso (Fig. 6). This is likely due to the injuries to organs and major arteries resulting in blood loss, which is a leading cause of shark-bite fatalities^29^. This is the first time that the location of a shark bite on the body has been assessed relative to recovery status.

Understanding how the location of a shark-bite wound relates to victim recovery has value in informing the development of shark-bite mitigations. For example, the development of shark-bite-resistant wetsuits could potentially result in higher survival rates of the user if the fabric is concentrated around the torso region^30,31^. Redesigning data acquisition and entry process to allow for categorical columns permits these types of analyses.

Some detail of a shark-bite incident might be lost by converting previously descriptive columns into categorical columns. We addressed this by complementing categorical columns with accompanying fields to retain details of the incident. For example, *injury location* and *injury severity* columns are both categorical and allow for user-friendly data analysis, whereas the *injury description* column is descriptive and provides added detail about the victim’s injuries if applicable. Individually, all three columns address certain aspects of the victim’s injuries, and together, all three columns comprehensively summarise injuries to the shark-bite victim.

Our analysis of the *Australian Shark-Incident Database* suggests that tiger sharks are proportionally responsible for the most fatalities of all shark species in Australia (38% of all tiger shark bites result in fatality), followed by bull sharks (32% of all bull shark bites result in fatality), and white sharks (25% of all white shark bites result in fatality) (Fig. 6). We emphasise that these figures represent the overall percentage of bites resulting in fatality since 1791 and do not account for possible changes over time. These figures are also proportional to the number of bites by each respective species. In Australia, white sharks are responsible for the largest number of bites on humans (361 total) compared to tiger (229 total) and bull sharks (197 total). At the time of publication, white and tiger sharks were each responsible for 91 and 86 total fatalities on humans in Australia, respectively.

**Fig. 7:**
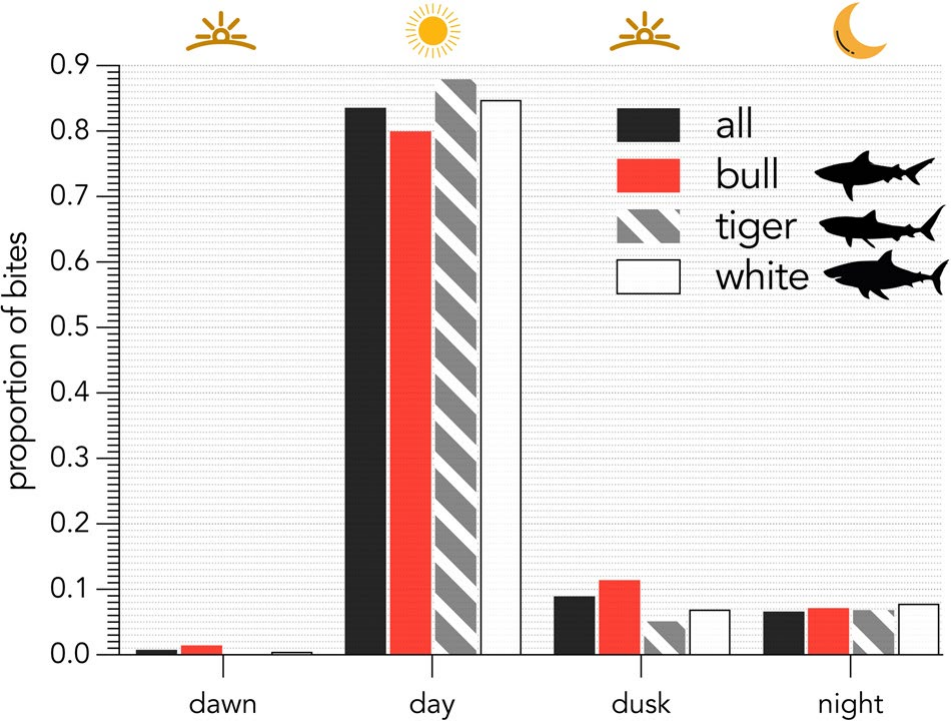
Period of day (corrected for local time) distribution of provoked and unprovoked shark bites in Australia by shark species from 1791 to 2022 (n = 540 bites from all species, 70 from bull, 59 from tiger, and 217 from white sharks).

There were 540 incidents in which time of day was recorded. We used these data to assess whether particular shark-bite incidents are more likely to occur at specific times of day (Fig. 7). To standardise reported 24-hour times, we took the location (latitude, longitude) information and reported time and date of each incident using the *getSunlightTimes* function in the suncalc library in R^32^ to calculate whether the incident occurred in one of four light-availability categories: *dawn*, *day*, *dusk*, or *night*. Shark bites occur mostly during the day, which likely reflects time of day when there are more ocean users present. However, there is a slightly higher proportion of bites at dusk for bull sharks compared to tiger and white sharks (Fig. 7). Identifying these trends can assist authorities in developing data-driven educational messaging as a shark-bite mitigation measure. This is important considering that enhanced education is the preferred mitigation measure scored by ocean users in New South Wales^33^.

These examples demonstrate that the *Australian Shark-Incident Database* will be useful for scientists to analyse environmental, social, and biological related shark-bite patterns in Australia. Use of the newly developed data descriptor to standardise future applications and account for quality assurance and control will aid in keeping the database consistent for ease of analysis and interpretation. Ultimately, the publishing of this database will improve our understanding of shark-bite incidents in Australia and will equip us with the knowledge to aim to avoid or predict these events in the future.

## Usage Notes

The database presented in this paper is publicly accessible online (10.5281/zenodo.5612259^26^). All names and personal information have been removed from the publicly available version. All data formats should remain consistent and are specified in Supplementary File 2. For example, shark-bite coordinates are presented as decimal degrees. The database will be updated with future incidents periodically, accordingly, figures and analyses presented in this paper could change as new data are added.

## Supplementary information

Supplementary files

## Data Availability

The R codes and packages used to create the plots in this paper can be accessed at github.com/cjabradshaw/AustralianSharkIncidentDatabase. These codes can be used to recreate summaries reported in this paper.
