# Supplementary material for: The Australian Shark-Incident Database for quantifying temporal and spatial patterns of shark-human conflict
Source: Sci Data. 2022 Jul 6;9:378. doi: 10.1038/s41597-022-01453-9 (PMC9259633; doi:10.1038/s41597-022-01453-9)
Supplement: Supplementary file 1 — Supplementary files [file 41597_2022_1453_MOESM1_ESM.pdf]

Table of Contents

Supplementary File 1: *Australian Shark-Incident Database* Questionnaire

Supplementary File 2: Description of the fields included in the *Australian Shark-Incident Database*

Supplementary File 1

AUSTRALIAN SHARK INCIDENT DATABASE QUESTIONNAIRE

Please fill in with as much detail as possible and return to:

Phoebe Meagher (ASID@zoo.nsw.gov.au)

Taronga Zoo, P.O. Box 20, Mosman. 2088 Ph # : 02 - 9978 4610

<u>Details of person completing form</u>

Surname: ………………………………

First name: …………………………….

Date: ……………………………………

Phone number (optional) …...………

Email address (optional): …………..

<u>Victim details</u>

Victim surname: ………………………

Victim first name: …………………….

Victim's phone number (optional)

…………………………………………….

Victim's email address (optional):

…………………………………………….

Victim gender (please circle):

Female, male, other ……………………

Victim age: ……………………………..

Incident date: …../…../……….

<u>Incident details</u>

Victim injury (please circle): Fatal, injured, uninjured

State incident occurred (please circle): WA, SA, VIC, NSW, QLD, NT, TAS

Location (closest town or beach):

……………………………………………

……………………………………………

Site category (please circle): River, estuary/harbour, coastal, ocean/pelagic, island open ocean (includes offshore shallow reefs), other

Shark common name, if known (if not known, provide description of shark):

……………………………………………

……………………………………………

Number of sharks present? …………

Victim activity (please circle):

- Snorkelling (includes freediving)
- Motorised boating
- Unmotorised boating (includes canoeing, kayaking or sailing)
- Boarding (includes surfboarding, bodyboarding, kiteboarding, sailboarding, wakeboarding and stand-up paddle boarding)
- Spearfishing
- Swimming (includes body surfing, clinging to an object, falling into water, floating or wading)
- Standing
- Diving (includes SCUBA diving, hookah diving or hard hat diving)
- Fishing (includes cleaning fish)
- Other……………………………… ……………………………………

If spearfishing, was a fish already speared? (either by the victim or others in the area, please circle): Yes, no

If applicable, provide additional detail of victim's activity ………………………… ……………………………………………………………………………………………… ……………………………………………………………………………………………… ………………………………………………………………………………………………

Was the victim undertaking commercial diving e.g., abalone collecting, crayfishing? (please circle): Yes, no

*Note: additional detail to be added in question above

If commercial diving, was catch already collected? (either by the victim or others in the area, please circle): Yes, no

What object was bitten? (if victim, please this field blank. Please circle):

- Board
- Dive equipment
- Catch (e.g., fish)
- Vessel (including canoe/kayak)
- Bait
- Other animal in area (e.g., schooling fish or dog)
- Other…………………………..…

Were any of these present at the time of the incident? (please circle):

- Fishing
- Spearfishing
- Bait and/or berley
- Catch
- Abattoir
- Baitfish
- Fish cleaning
- Commotion/splashing
- Dead animal nearby (e.g., whale)
- Discharge site (e.g., rivermouth, desalination plant)
- Other sealife (e.g., seals, turtles, dolphins) …………….
- Boating
- Swimming
- Boarding
- Snorkelling
- Scuba-diving
- Freediving
- Other……….…….….….….……
……….……….….……….….…
………………………………..

Direction at which shark first made contact with the victim e.g., first bite, initial bump (please circle):

- Above
- Below
- Behind
- In front
- Left
- Right

Describe the shark's behaviour ………………………………………………………
…………………………………………………………………………………………….
…………………………………………………………………………………………….

Was the victim aware of the shark prior to the incident? (please circle): Yes, no

Where was the victim injured by the shark? (please circle all that apply):

- Hand
- Lower arm
- Upper arm
- Shoulder
- Neck
- Head
- Torso
- Foot
- Calf
- Thigh
- Pelvic region
- Other……………………….….…

Indicate the severity of the victim's injuries that were sustained by the shark:

- Surface wound (e.g., bruising)
- Abrasion (removal of skin e.g., grazes/scratches)
- Punctures
- Minor lacerations (e.g., shark has punctured and torn skin and soft tissue; loss of function is not seen)
- Major lacerations (e.g., shark has punctured and torn skin, muscle, tendon, or bone; loss of function seen; surgical procedures required)
- Bone breakages (including fractures)
- Injuries to internal organs (e.g., ruptured lung)
- Amputation (i.e. loss of limb)
- Other……………………………….. ………………………………………. ……………………………………

Describe the victim's injury in more detail ……………………………………………
……………………………………………………………………………………………….
……………………………………………………………………………………………….
……………………………………………………………………………………………….

What was the victim was wearing at the time of the incident? (please circle):

- Clothes
- Swimwear
- Wetsuit/drysuit
- Shark-proof suit
- Nothing
- Other…………………………… …………………………………… ……………………………….

How much body coverage did the victim's clothing provide? (please circle):

- Full body
- Full arm-short legs
- Short arm-full legs
- Short arm-short legs
- Short only
- Single-piece
- Bikini
- Speedo

What was the dominant colour of the victim's clothing/swimwear? (accounting for at least 50% of overall colour, please circle):

- Black
- Grey
- White
- Blue
- Yellow
- Orange
- Red
- Green
- Pink
- Purple
- Brown
- Metallic
- Multi-colour ……………………
  Other (e.g. patterned) ………..
  …………………………………..

If applicable, what was the main colour on the victim's fins? (please circle):

- Black
- Grey
- White
- Blue
- Yellow
- Orange
- Red
- Green
- Pink
- Purple
- Brown
- Metallic
- Translucent
- Multi-colour ……………………...
- Other………………………………
  ……………………………….

If applicable, describe how the victim or others took action to divert the shark

……………………………………………………………………………………………
……………………………………………………………………………………………

What was the effect of the diversionary action taken? (please circle):

- No effect
- Effective
- Shark bite
- Shark released victim momentarily then renewed attack
- Shark released victim permanently
- Shark left the area
- Shark killed
- Shark kept at a distance
- Shark followed
- Other……………………………

Please provide the name, phone number, email address and/or address of any witnesses

……………..…………..…………..…………..…………..…………..…………..……….

……………..…………..…………..…………..…………..…………..…………..……….

Number of people within 3 meters of the incident?

…………… people

Number of people between 3 and 15 meters of the incident?

…………… people

What was the time of day at which the incident occurred? Please use 24-hour time (e.g., 14:00) ……………

If not on the surface, what depth did the bite take place? (not total water depth)

…………… meters

Were any shark teeth or teeth fragments found in the victim or the victim's equipment? (please circle): Yes, no

Estimated time the victim was in the water prior to the incident: ………. minutes

Temperature of water at the time and place of the incident: ………. °C

Total depth at which the incident took place (surface to sea/river floor) ……………. meters

Estimated water clarity at the area and time at which the incident took place:

…………… meters

Distance from where the incident took place to the shore: …………… meters

Tidal cycle at time of incident (please circle):

- Low
- Mid
- High

What were the wind conditions? (please circle):

- Calm
- Light breeze
- Moderate breeze
- Windy

State of the weather at the time of the incident (please circle all that apply):

- Sunny
- Cloud cover
- Overcast
- Rainy
- Stormy
- Other……..………………………
  ……………………………………
  ……………………………………

Ambient air temperature at the time and place of the incident: ………. °C

Indicate whether the victim was using protective equipment during the incident (please circle all that apply):

- Speargun
- Knife
- Board
- Shark suit
- Deterrent/repellent
- Other……..………………………
  ……………………………………
  ……………………………………

If a deterrent/repellent was used, describe the *brand*, *make*, *model*, and whether it was believed to be in effect/switched *on or off*:

…..……..……..……..……..……..……..……..……..……..……..……..……..……..

……..……..……..……..……..……..……..……..……..……..……..……..……..……..

……..……..……..……..……..……..……..……..……..……..……..……..……..…

Are photos available and filed of the shark, victim, or scene of the incident? (please circle): Yes, No

Please detail any other comments or information relevant to the incident:

…..……..……..……..……..……..……..……..……..……..……..……..……..…….

……..……..……..……..……..……..……..……..……..……..……..……..……..……

…..……..……..……..……..……..……..……..……..……..……..……..……..…….

……..……..……..……..……..……..……..……..……..……..……..……..……..……

…..……..……..……..……..……..……..……..……..……..……..……..……..…….

……..……..……..……..……..……..……..……..……..……..……..……..……..……

…..……..……..……..……..……..……..……..……..……..……..……..……..…….

………..……..……..……..……..……..……..……..……..……..

How do you feel about lethal shark control (e.g., culling, shark nets)? (please circle)

- I support these measures
- I oppose these measures

There are many different circumstances and attitudes towards shark encounters. What best describes your experience and attitudes?

I was aware of previous human-shark encounters at this location
Strongly agree *Agree* *Neutral* *Disagree* *Strongly disagree*

I believed the risk of shark encounters was high at this location
Strongly agree *Agree* *Neutral* *Disagree* *Strongly disagree*

Before entering the water I was concerned about an encounter with a shark at this location
Strongly agree *Agree* *Neutral* *Disagree* *Strongly disagree*

You can tell when water conditions are more 'sharky'
Strongly agree *Agree* *Neutral* *Disagree* *Strongly disagree*

SIGNATURE .... **Prepared by ....**

Date report prepared ..../……..../.........

I give permission for the Australian Shark Bite File to contact my Doctor and release relevant details of the injuries sustained (if required).

SIGNATURE ....

For office use only

Latitude (Decimal degrees e.g. -33.8546) ..…..°

Longitude (Decimal degrees e.g. 151.2168°) ……..°

Shark ID method (please circle):

- Footage
- Tooth recovered
- Bite analysis
- Shark behaviour
- DNA analysis
- Geographical location
- Direct observation
- Other………………………………
  …………………………………

Shark ID source (please circle):

- Witness/victim
- Footage
- Government official
- Researcher
- Other……………………………………………………………

Was a shark captured after the incident? (please circle): Yes, no

Indicate the tide type at the time of the incident (please circle):

- Spring (soon after a new or full moon)
- Neap (soon after the first or third quarters of the moon)

Australian Shark-Incident Database

REPORTING A HUMAN-SHARK INTERACTION

The Australian Shark-Incident Database is appreciative of any assistance you can give by filling out the Questionnaire as accurately as possible. The information provided or uncovered about a shark attack, historic or new, will be managed in a confidential manner. Data will be used to analyse the circumstances surrounding the event for scientific purposes. Personal detail (name & addresses, etc) are not given out to media. The media is explicitly barred from direct access to ASID cases files.

If multiple parties witnessed an incident, we would appreciate it if you would ask them also to fill out a copy of the questionnaire independently so that we can get their perspective on the incident. Inclusion of any corroborative documentation (such as newspaper articles, police, medical reports, photographs of the victim, gear worn by the victim, and the attack site; etc.) is also most appreciated, as is environmental information (e.g., water temperature, water salinity, tidal state, habitat, etc.). The goal is to gather as much information as possible relevant to the attack.

We are interested in documenting the medical aspects of a bite to assist in accurate species identification. If possible, we would welcome photographs of the injuries and the permission to request copies of relevant medical records.

Information from this questionnaire may be used by professional researchers. Identifiers such as names and contact details will not be shared publicly to provide anonymity and confidentially to victims. For any further information on this please contact Taronga Conservation Society Australia.

All information will also be available to the International Shark Attack File for public education, statistical research on a worldwide basis.

Thank you for your participation.

Rodd Stapley
Curator, Australian Shark-Incident Database

Supplementary File 2

Description of the fields included in the *Australian Shark-Incident Database*, used as a protocol for data entry into the database.

Column name	Description	Values or units
UIN	unique identifying number. To be added in consecutive order.	numeric
ISAF.no	reference number in *International Shark Attack File*	numeric
Details.of.person.completing.form	name, date, phone and email address of person filling out questionnaire	descriptive
Last.name	victim surname only; capitalised first letter	descriptive
First.name	victim first name only; capitalised first letter	descriptive
Victim.contact.details	name, date, phone and email address of the shark-bite victim	descriptive
Incident.day	day of month	numeric, integer
Incident.month	month of year	numeric, integer
Incident.year	year written in full	numeric, integer, *yyyy* e.g., 2007, not 07
Victim.injury	outcome of victim's health. Categories: fatal, injured, or uninjured. Fatal bites include bites resulting in death to the victim. Injured bites include bites resulting in physical injury to the victim (e.g., bruising, abrasion, punctures, lacerations). Uninjured bites include interactions resulting in no injury to the victim (e.g., shark bit the victim's equipment; surfboard, fishing rod, kayak).	categorical
State	Australian State/Territory; abbreviated; categories: *WA, SA, VIC, NSW, QLD, NT, TAS*	categorical
Location	closest town and/or beach	descriptive
Latitude	latitude of incident	numeric, decimal degrees
Longitude	longitude of incident	numeric, decimal degrees
Site.category	type of site where incident occurred; categories: *river, estuary/harbour, coastal, ocean/pelagic, island open ocean* (includes offshore shallow reefs), *other*; if other write: *other: detail*	categorical
Site.category.comment	any other relevant information regarding site description	descriptive
Shark.common.name	if identified to species, species common name; if identified to family, family group common name; common name should be Australian common names (Last and Stevens 2009); all lowercase, e.g., *requiem shark*	
Shark.scientific.name	scientific name following CSIRO Codes for Australian Aquatic Biota; capitalise first word only; e.g., *Carcharodon carcharias*	

Shark.identification.method	what information used to identify species; categories: t*ooth recovered, bite analysis, shark behaviour, DNA analysis, geographical location, direct observation, other*; if multiple categories relevant separate by comma; if *other* write: *other: detail*	categorical
Shark.identification.source	source for species identification; categories: *witness/victim, government official, researcher, footage, other*; if multiple categories relevant separate by comma; if *other* write: *other: detail*	categorical
Shark.length.m	estimated length of shark	numeric, metres, one decimal point i.e., 3.5
Basis.for.length	what information used to estimate shark length; categories: *witness/victim, government official, researcher, footage, bite radius estimation, other*; if multiple categories relevant separate by comma; if *other* write: *other: detail*	categorical
Provoked.unprovoked	categories: *provoked, unprovoked*. *Unprovoked* is defined as an encounter between a human and a shark where a shark is in its natural habitat and has made a determined attempt to bite a human where that person is not engaged in provocative activities. *Provoked* is defined as an encounter between a human and a shark where the person attracts or initiates physical contact with a shark (accidentally or on purpose) or was fishing for, stabbing, feeding, netting, or handling a shark, or where the shark was attracted to the victim by activities such as fishing, spearfishing (where a fish has already been speared), commercial diving (e.g., collecting abalone, pearl shells, or other marine animals where catch has already been collected), and cleaning of captured fish.	categorical
Provocative.act	if *provoked*, basis of provocation; categories: *physical contact* (i.e., victim poked, stood on, held, pushed shark), *enticed shark* (i.e., cleaning, collecting, fishing, or feeding other sea life), *capture of shark*, *victim moved into immediate proximity of shark, victim fed shark, victim intentionally hurt shark* (e.g., stabbed shark), *other*; if multiple categories relevant separate by comma; if *other* write: *other: detail*	categorical
No.sharks	number sharks present at time of incident	numeric
Victim.activity	activity at time of incident; categories: *snorkelling, motorised boating, unmotorised boating, boarding, spearfishing, swimming, diving, fishing, other.* unmotorised boating includes canoeing, kayaking, sailing; snorkelling includes freediving; boarding includes surfboarding, bodyboarding, kiteboarding, sailboarding, wakeboarding, stand-up paddle boarding; swimming includes body surfing, clinging to object,	categorical

	falling into water, floating, or wading; diving includes scuba, hookah, hard-hat diving; fishing includes cleaning fish; if *other* write: *other: detail*	
Fish.speared?	if the activity was spearfishing, indicate whether a fish had already been speared (either by the victim or someone in the vicinity); categories: Y, N	categorical
Victim.activity.details	additional detail of victim's activity (e.g., type of boarding undertaken – surfboarding, stand up paddle boarding, kiteboarding etc.), if applicable.	descriptive
Commercial.dive.activity	victim commercial diving? e.g., abalone collecting, crayfishing; additional detail added to 'Victim Activity Detail'; categories: *Y, N*	categorical
Object.of.bite	object shark was primarily motivated to bite (excluding victim); categories: *board, dive equipment, catch* (fish), *vessel* (including canoe/kayak), *bait, other animal in area* (e.g., schooling fish or dog), *other*; if victim, leave field blank; if multiple categories relevant separate by comma; if *other* write: *other: detail*	categorical
Present.at.time.of.bite	other activities present in area; categories: *boating, swimming, boarding, snorkelling, scuba-diving, freediving, fishing, spearfishing, bait and/or berley, catch, abattoir, baitfish, fish cleaning, commotion/splashing, dead animal nearby* (e.g., whale), *discharge site* (e.g., river mouth, desalination plant), *other sea life* (e.g., seals, turtles, dolphins; specify in bracket), *other*; if multiple categories relevant separate by comma; if *other* write: *other: detail*	categorical
Present.at.time.of.bite.detail	provide detail about other activities present; e.g., crayfish bagged, grabbed shark by tail	descriptive
Direction.first.strike	direction from which shark first made contact with victim, e.g., first bite, initial bump; categories: *above, below, behind, in front, left, right*; if multiple categories relevant separate by comma	categorical
Shark.behaviour	describe the shark's behaviour prior, during and/or after the incident took place	descriptive
Victim.aware.of.shark	whether victim was aware of shark prior to incident; categories: *Y, N*	categorical
Shark.captured	whether shark was captured after incident; categories: *Y, N*	categorical

Injury.location	which areas on victim injured by shark; categories: *arm, hand, lower arm, upper arm, shoulder, neck, head, torso, leg, foot, calf, thigh, pelvic region, other*; if multiple categories relevant separate by comma; if *other* write: *other: detail*	categorical
Injury.severity	severity of victim's injuries sustained from shark; categories: *bruising, abrasion* (removal of skin, i.e., grazes/scratches), *punctures, minor lacerations* (i.e., punctured/torn skin and soft tissue; loss of function is not seen), *major lacerations* (i.e., punctured/torn skin, muscle, tendon, or bone; loss of function seen; surgical procedures required), *bone break* (including fractures), *injuries to internal organs* (e.g., ruptured lung), *amputation, other*; if multiple categories relevant separate by comma; if *other* write: *other: detail*	categorical
Injury.description	describe victim's injury in more detail if applicable	descriptive
Victim.gender	categories: *female, male, other*; if multiple categories relevant separate by comma; if *other* write: *other: detail*	categorical
Victim.age	age of victim in years	numeric, integer, years
Victim.clothing	what victim was wearing at time of incident; categories: *clothes, swimwear, wetsuit/drysuit, shark-proof suit, none, other*; if *other* write: *other: detail*	categorical
Clothing.coverage	% body coverage victim's clothing provided; categories: *full body, full arm-short legs, short arm-full legs, short arm-short legs, short only, one-piece, bikini, speedo*	categorical
Dominant.clothing.colour	clothing colour accounting for > 50% overall colour; categories: *black, grey, white, blue, yellow, orange, red, green, pink, purple, brown, metallic, multicolour, other*; if two colours dominant (i.e., 50% each) write: *multicolour: colour, colour*; if *other* write: *other: detail*	categorical
Other.clothing.colour	other colour/s present on clothing; categories: *black, grey, white, blue, yellow, orange, red, green, pink, purple, brown, multicolour, other*; if multiple categories relevant separate by comma; if *other* write: *other: detail*	categorical
Clothing.pattern	any dominant pattern/s on victim's clothing; categories: *block colour, camouflage, stripe, checkered, swirl, floral, speckled, other*; if multiple categories relevant separate by comma; if *other* write: *other: detail*	categorical
Fin.colour	colour/s on victim's fins/flippers; categories: *black, grey, white, blue, yellow, orange, red, green, pink, purple, brown, metallic, multicolour, translucent, other*; if two colours dominant (i.e., 50 % each) write *multicolour*: colour, colour; if *other* write: *other: detail*	categorical
Diversionary.action.taken	if applicable, how victim or others took action to divert shark	descriptive

Diversionary.action.outcome	effect of diversionary action taken; categories: *no effect, effective, shark bite, shark released victim momentarily then renewed attack, shark released victim permanently, shark left the area, shark killed, shark kept at a distance, shark followed, other*; if multiple categories relevant separate by comma; if *other* write: *other: detail*	categorical
Witness.details	witness name, contact details and comment of witness	descriptive
People < 3 m	number of people within 3 metres of incident	numeric, integer
People 3–15 m	number of people between 3 and 15 m of incident	numeric, integer
Time.of.incident	time of day incident occurred	numeric, 24-hour time; no colon i.e., 1830
Depth.of.incident.m	estimated depth at which the shark bite took place (not total water depth)	numeric, metres, to one decimal point i.e., 3.5
Remains.in.shark	whether victim's remains found in shark after incident; categories: *arm, hand, lower arm, upper arm, shoulder, neck, head, torso, leg, foot, calf, thigh, genitalia, other*; if multiple categories relevant separate by comma; if *other* write: *other: detail*	categorical
Teeth.recovered	whether any shark teeth or teeth fragments found in victim or victim's equipment; categories: *Y, N*	categorical
Time.in.water.min	estimated time victim was in water prior to incident	numeric, minutes
Water.temperature.°C	temperature of water at time and place of incident	numeric, °C, one decimal point i.e., 15.5
Total.water.depth.m	estimated total depth (surface to sea/river floor) at which incident took place	numeric, metres, to one decimal point i.e., 3.5
Water.visability.m	estimated water clarity at area and time at which incident took place	numeric, metres, to one decimal point i.e., 3.5
Distance.to.shore.m	estimated distance from where incident took place to closest land mass	numeric, metres, to one decimal point i.e., 50.5
Spring.or.neap.tide	tide type at time of incident; categories: *spring, neap*	categorical
Tidal.cycle	tidal cycle at time of incident; categories: *low, mid, high*	categorical
Wind.condition	Beaufort wind force scale of sea state at time of incident; categories: *calm, light breeze, moderate breeze, windy*	categorical
Weather.condition	state of weather at time of incident; categories: *sunny, cloud cover, overcast, rainy, stormy, other*; if multiple categories relevant then separate by comma; if *other* write: *other: detail*	categorical
Air.temperature.°C	ambient air temperature at time and place of incident	numeric, °C, to one decimal point i.e., 15.5
Personal.protective.device	whether the victim was using protective equipment during incident; categories: *speargun, deterrent/repellent, knife, board, shark suit, other*; if multiple categories relevant then separate by comma; if *other* write: *other: detail*	categorical
Deterrent.brand.and.type	if deterrent/repellent used, brand, make, model, and whether was in effect/switched on/off	descriptive

Photos	photos available and filed of shark, victim, or scene of incident? categories: *Y, N*	categorical
Data.source	information used to compile this record; selected categories should be direct account from source (e.g., victim interview in newspaper article should be categorised as *media outlet* not *victim account*); categories: *ASID questionnaire, other questionnaire (specify in brackets), victim account, witness account, other person account, government report, expert* (e.g., researcher, medical professional), *book, media outlet, footage, other*; if multiple categories relevant then separate by comma; if *other* write: *other: detail*	categorical
Comment	any other comments or information relevant to incident	descriptive
Reference	reference for data source if applicable, e.g., newspaper publication title and date	descriptive
